# Sex differences in relationships between metabolic syndrome components and factors associated with health-related quality of life in middle-aged adults living in the community: a cross-sectional study in Taiwan

**DOI:** 10.1186/s12955-018-0910-2

**Published:** 2018-04-27

**Authors:** Cheng-Chieh Liu, Hsiao-Ting Chang, Shu-Chiung Chiang, Harn-Shen Chen, Ming-Hwai Lin, Tzeng-Ji Chen, Shinn-Jang Hwang

**Affiliations:** 1 Division of Family Medicine, Department of Medicine, Kaohsiung Veterans General Hospital Pingtung Branch, No.1, Anping Lane 1, Jausheng Rd, Neipu Township, Pingtung Taiwan; 2 Department of Family Medicine, Taipei Veterans General Hospital, No. 201, Section 2, Shipai Road, Beitou District, Taipei, Taiwan; 3 School of Medicine, National Yang-Ming University, Taipei, Taiwan; 4 Institute of Hospital and Health Care Administration, National Yang-Ming University, Taipei, Taiwan; 5 Division of Endocrinology and Metabolism, Department of Medicine, Taipei Veterans General Hospital, No. 201, Sec. 2, Shipai Road, Beitou District, Taipei, Taiwan

**Keywords:** Health-related quality of life, Metabolic syndrome, Physical activity, Sex differences, SF-36

## Abstract

**Background:**

Metabolic syndrome (MetS) is a widespread condition with important effects on public health, in general. There is a lack of relevant research on possible sex differences in the relationship between MetS and health-related quality of life (HRQoL) and also the sex differences in factors associated with HRQoL. The aims of this study were to identify: 1) whether women exhibit greater negative impacts on physical domain HRQoL from MetS compared with men; 2) whether women exhibit greater mental domain impacts compared with men; and 3) whether factors associated with HRQoL scores are different for men and women.

**Methods:**

This cross-sectional study was conducted in Taipei, Taiwan. Using random sampling, a total of 906 participants aged 35–55 years were recruited. MetS was defined according to the MetS criteria for the Taiwanese population, and HRQoL were assessed using physical component summary (PCS) and mental component summary (MCS) scores of the Short Form Health Survey (SF-36), Taiwan version. Demographics, physical activity, medical history, and blood tests as covariates were recorded and checked. The associations were assessed by multiple linear regression.

**Results:**

After adjusting for covariates, women but not men with more components of MetS had significantly lower PCS scores (β = − 0.542, *p* = 0.036). The number of components of MetS was not a significant factor in MCS score differences between the sexes. Furthermore, there were sex differences regarding age, education level, physical activity, and smoking status in association with PCS scores. For MCS scores, sex differences were found in education level, marital status, and habits of smoking and alcohol consumption.

**Conclusions:**

There were sex differences in the relationships between metabolic syndrome components and factors associated with HRQoL among middle-aged adults living in the community in Taiwan. Further research should be conducted to investigate mechanisms of these sex differences.

## Background

The term metabolic syndrome (MetS) refers to a collection of conditions, including central obesity, elevated triglyceride, elevated blood pressure, fasting hyperglycemia, and low levels of high-density lipoprotein cholesterol (HDL-C). MetS is regarded as a major public health issue in many developed and developing countries [1]. Many studies have shown that MetS is associated with increased risks of diabetes, cardiovascular diseases, cerebrovascular diseases, poorer quality of life, and mortality [2–5]. According to the 1999–2000 National Health and Examination Survey (NHANES) in the United States, the age-adjusted prevalence of MetS was 27.0%, according to Adult Treatment Panel III (ATP III) criteria [6]. A systemic review conducted in 2013 showed that the weighted mean general prevalence of MetS in Brazil was 29.6% [7]. In mainland China, a meta-analysis of 35 studies that collectively analyzed data from 2000 to 2015 for 2,26,653 Chinese participants more than 15 years old found that the pooled prevalence of MetS was 24.5% [8]. In Taiwan, according to the 2005–2008 Nutrition and Health Survey in Taiwan [9], the prevalence of MetS among all people over 19 years of age was 28.5%; the prevalence in women was 31.5% and it was 25.5% in men, with prevalence generally increasing with age. According to a 2005 study, the overall prevalence of MetS in Japan was 5.3%, with rates of 4.4% among women and 6.2% among men. The low rate of MetS in Japan might be related to the fact that the Japanese population generally consumes a balanced diet, engages in brisk walking and regular exercise, and works 3 to 7 h a day [10]. The prevalence of MetS in Taiwan is therefore similar to rates in the United States, Brazil, and mainland China and are substantially higher than those in Japan. Furthermore, there is an increasing prevalence of MetS in the middle-aged population of Taiwan (i.e., those aged 30–65 years), especially men [11].

Health-related quality of life (HRQoL) is a broad, multidimensional concept that usually includes subjective evaluations of both positive and negative aspects of life [12]. Health has traditionally been measured narrowly and from a deficit perspective, often using measures of morbidity or mortality. Owing to advances in modern medicine and improved social welfare, however, average human life expectancy has increased. At the same time, people have gradually noted that health is no longer viewed as purely a matter of longevity but is now also seen as a multidimensional construct that includes physical, mental, and social domains [13]. As such, the ways in which health outcomes are measured should assess the health of a population not only with the aim of saving lives but also to improve quality of life among the population.

Previous studies on associations between MetS and HRQoL have found that participants who exhibit more components of MetS generally have a poorer quality of life [14, 15]. Regarding sex differences between the association of MetS components and HRQoL, there have been few studies that have considered differing HRQoL impacts among men and women. A previous study of the general population in Iran indicated that MS is associated with poor HRQoL in women but not in men, and that this association is formed mainly in relation to physical rather than mental health [14]. Another study in Korea found that MS has a significant negative impact on the HRQoL of middle-aged Korean women but no significant impact on that of middle-aged Korean men [16]. The negative impacts of MetS on women might be related to several factors, including the level of physical morbidity, the prevalence of chronic conditions, and the level of pain [16]. Another study in Japan examining relationships between HRQoL and clustering of MS diagnostic components in participants aged 19–69 years found that the number of components of MetS was negatively associated with the general health score of HRQoL but positively associated with the mental health score of HRQoL in both men and women [15].

Given that MetS is a widespread condition with important public health effects in general, and considering the lack of relevant research on possible sex differences in the relationship between MetS and HRQoL, and also sex differences in the factors associated with HRQoL, the aim of this study was to identify: 1) whether women exhibit greater negative impacts on physical domain HRQoL owing to MetS compared with men; 2) whether women exhibit greater mental domain impacts compared with men; and 3) whether factors associated with HRQoL scores differ between men and women.

## Methods

### Ethical statement

This study was approved by the institutional review board of Taipei Veterans General Hospital, Taipei, Taiwan. Informed consent was obtained from all individuals who participated in the study.

### Study design, target population, and participants

This study was a cross-sectional study conducted in nine neighborhoods in the Shipai area of Taipei, Taiwan during 2009–2010. The inclusion criteria were as follows: (1) residents of Shipai who had lived in the area for at least 6 months, and (2) aged between 35 and 55 years. Potentially eligible participants were identified from the official government household registration system in December 2008 and January 2009. Those who fulfilled with the inclusion criteria were randomly sampled and were sent invitation letters. Individuals who agreed to participate were included after they had signed an informed consent form. Participants then underwent examination and answered questionnaires at the research center of Taipei Veterans General Hospital. The response rate was 10.1%. A total of 906 adults participated in this study; of these, 61.7% were female. The mean age of all participants was 46.9 ± 5.5 years, and there was no significant age difference between male and female participants. The mean BMI of participants was 23.8 ± 3.5 kg/m^2^, and mean waist circumference was 83.4 ± 9.9 cm. A total 37.5% of male participants had waist circumference ≥ 90 cm, and 44.5% of female participants had waist circumference ≥ 80 cm. Compared with women, men had a lower rate of illiteracy or elementary school education (0.9% vs. 2.3%) and a higher rate of university education level and above (69.7% vs. 57.7%, *p* = 0.001). A higher percentage of male participants than female ones were married (89.6% vs. 82.0%, *p* = 0.002). There were also significant differences between men and women in terms of smoking status and alcohol consumption. More male participants were current or ex-smokers and current drinkers. With respect to physical activity, there was no significant difference in physical activity levels between male and female participants (*p* = 0.866) (Table 1).

**Table 1:** Demographic characteristics of participants

	Total		Men		Women		*P* value
	*n*	%	*n*	%	*n*	%	
Sex			–	–	–	–	–
Men	347	38.3	–	–	–	–	–
Women	559	61.7	–	–	–	–	–
Age (mean ± SD)	46.9	5.5	46.9	5.7	46.9	5.5	0.884
BMI (mean ± SD)	23.8	3.5	25.2	3.3	23	3.4	< 0.001
BMI							< 0.001
< 18.5	30	3.3	5	1.4	25	4.5	
18.5–23.9	492	54.3	129	37.2	363	64.9	
≥ 24	384	42.4	213	61.4	171	30.6	
Waist circumference (mean ± SD)	83.4	9.9	88.4	8.7	80.3	9.3	< 0.001
< 80 cm	–	–	–	–	310	55.5	–
≥ 80 cm	–	–	–	–	249	44.5	
< 90 cm	–	–	217	62.2	–	–	
≥ 90 cm	–	–	130	37.5	–	–	
Education level							0.001
Illiterate/Elementary school	16	1.8	3	0.9	13	2.3	
Senior / Junior high school	324	35.8	102	29.4	222	39.9	
University and above	563	62.1	242	69.7	321	57.7	
Marital status							0.002
Married	764	84.3	309	89.6	455	82.0	
Single/Divorced/Separated/ Widowed/others	136	15	36	10.4	100	18.0	
Personal habits							
Smoking							< 0.001
Non-smoker	723	79.8	205	59.2	518	93.0	
Current smoker	125	13.8	96	27.7	29	5.2	
Ex-smoker	55	6.1	45	13.0	10	1.8	
Alcohol consumption							< 0.001
No	765	84.4	240	69.2	525	94.3	
Yes	139	15.3	107	30.8	32	5.7	
Physical activity (IPAQ score)							0.866
Low	318	35.1	120	34.6	198	35.4	
Moderate	403	44.5	153	44.1	250	44.7	
High	185	20.4	74	21.3	111	19.9	

### **Definitions of components of M**et**S**

The five components of MetS considered in this study were chosen according to the criteria proposed by the Taiwan National Department of Health in 2007, as follows: (1) central obesity, defined as a waist circumference ≥ 90 cm for men and ≥ 80 cm for women; (2) elevated blood pressure, defined as systolic blood pressure ≥ 130 mmHg, diastolic blood pressure ≥ 85 mmHg, or receiving any specific treatment for hypertension; (3) elevated triglyceride, defined as triglyceride ≥150 mg/dL or receiving any specific treatment for this lipid abnormality; (4) low HDL-C, defined as HDL-C < 40 mg/dL for males, HDL-C < 50 mg/dL for females, or receiving any specific treatment for this lipid abnormality; and (5) fasting hyperglycemia, defined as fasting plasma glucose ≥100 mg/dL or previously diagnosed type 2 diabetes [17]. If a person had three or more of the above five components, then they were regarded as having MetS. Accordingly, the number of MetS components for each participant in this study was recorded and subjected to further analysis.

### Measurement of HRQoL

The Short Form Health Survey (SF-36), Taiwan version, which was developed following International Quality of Life Assessment (IQOLA) project protocols [18, 19], was used for evaluating HRQoL among participants in this study. This questionnaire was self-administered by participants with the help of trained research assistants at the research center of Taipei Veterans General Hospital. The SF-36 measures eight health domains that can be grouped into two categories under the physical component summary (PCS) and mental component summary (MCS), with overall PCS and MCS scores representing subjective physical health and mental health, respectively. The PCS comprises the four physical health domains of the SF-36: physical functioning (ten items), role limitations owing to physical problems (four items), bodily pain (two items), and general health (five items). The MCS comprises the other four domains, which are mental health domains: vitality (four items), social functioning (two items), role limitations owing to emotional problems (three items), and mental health (five items). Another single item evaluating perceived change in health is also included in the SF-36. Responses to these 36 items are scored according to the scoring instructions provided in the SF-36 Health Survey Manual and Interpretation Guide. For each domain, the score ranges from 0 to 100, with lower scores indicating more impaired HRQoL. Previous studies revealed that the SF-36 Taiwan version has good validity and reliability with a Cronbach’s α of 0.8 [18, 19]. The Cronbach’s α of SF-36 in this study was 0.68.

### Demographics, medical history, medications, and physical activity

For each participant, demographic characteristics including sex, age, educational level, marital status, personal habits of cigarette smoking and alcohol consumption, and medical history were recorded using participants’ reports. Reported educational level was categorized as illiterate/elementary school, senior/junior high school, or university and above. Reported marital status was categorized as married or single/divorced/separated/widowed/other. Key aspects of participants’ self-reported medical history, including diabetes mellitus, hypertension, and dyslipidemia, were recorded. The medications currently used, including antihypertensive agents, antidiabetic medication (insulin or oral agents), and lipid-lowering agents, were noted.

The International Physical Activity Questionnaire (IPAQ) Short-Form, Taiwan version, was used to measure physical activity in this study. Previous studies have reported good validity and reliability of this measurement in Taiwan [20]. This questionnaire was also self-administered by participants with the help of trained research assistants. The questionnaire consists of seven questions that measure the frequency and/or duration with which a person has engaged in vigorous physical activity, moderate physical activity, walking for at least 10 min at a time, or sitting, during the previous 7 days. The frequency and/or duration of each specific type of physical activity was computed by weighting its equivalent metabolic equivalent of task (MET), an energy expenditure indicator, to yield an IPAQ score in MET-minutes. The sum of MET-minutes, in turn, represents the overall volume of activity. Vigorous physical activity refers to activities with a value of 8 METs that require intense physical effort and make the person breathe much harder than normal; moderate physical activities refer to activities with a value of 4 METs that require moderate physical effort and make the person breathe somewhat harder than normal; walking is calculated as having a value of 3.3 METs. Accordingly, participants were stratified into three levels of physical activity according to their overall volume and frequency of activity: low, moderate, and high. A high level of physical activity refers to: (1) doing vigorous physical activities for at least 3 days, adding up to at least 1500 MET-minutes/week or (2) doing a combination of vigorous physical activities, moderate physical activities, and/or walking for at least 7 days adding up to at least 3000 MET-minutes/week. A moderate level of physical activity refers to: (1) doing vigorous physical activities for at least 20 min per day for at least 3 days, (2) doing moderate physical activities or walking for at least 30 min per day for at least 5 days, or (3) doing a combination of vigorous physical activities, moderate physical activities, or walking for at least 5 days adding up to at least 600 MET-minutes/week. A low level of physical activity refers to any level of activity not meeting the above criteria for high and moderate levels.

### Anthropometry, blood pressure, and blood tests

The body weight, height, body mass index (BMI), waist circumference, and systolic and diastolic blood pressure of each participant were measured and recorded. Height and weight were evaluated using an automatic electronic measuring device, and BMI was obtained via calculation. Waist circumference was measured by the standard method. Blood pressure was measured using a standardized sphygmomanometer applied with the participant in a seated position, after 30 min of rest.

Each participant fasted for at least 8 h before the collection of blood samples. Blood tests including fasting plasma glucose, triglycerides, and HDL-C were conducted at the central laboratory of Taipei Veterans General Hospital, which has obtained laboratory accreditation/certification from the College of American Pathologists.

### Statistical analysis

Statistical analyses were performed using Microsoft Excel (2016) and IBM SPSS, version 20.0 (IBM Corporation, Armonk, NY, USA). All analyses were stratified by sex. Descriptive statistics are presented as means and standard deviations for continuous variables, and as numbers and percentage for categorical variables. The differences between means were evaluated using a Student *t*-test (continuous variables with 2 categories) or analysis of variance (ANOVA) (continuous variables with ≥3 categories); the differences between categorical variables were evaluated using the chi-square test. Comparisons of HRQoL scores across physical activity groups were performed by ANOVA. Multiple linear regression models were used to determine the associations between covariates and PCS and MCS quality of life scores for all male and female participants. Covariates entered in the models included sex, age, education level, marital status, number of MetS components, physical activity level, and personal habits of smoking and alcohol consumption. For education level, we coded illiterate/elementary school as the reference level whereas for physical activity, we coded low physical activity as the reference level. A two-tailed *p* value < 0.05 was considered statistically significant.

## Results

Table 2 shows the sex differences in the components of MetS and SF-36 scores. Men had significantly higher proportions of high blood pressure compared with women (both systolic BP [47.8% vs. 21.6%, *p* <  0.001] and diastolic BP [40.3% vs. 16.8%, *p* <  0.001]). A significantly higher proportion of male participants demonstrated abnormal triglyceride levels (34.3% vs. 14.9%, *p* <  0.001) and fasting hyperglycemia than female participants (24.8% vs. 15.4%, *p* <  0.001). A significantly higher percentage of women had a large waist circumference (49.2% vs. 40.9%, *p* = 0.015). In terms of SF-36 scores, female participants had significantly lower scores than male ones in physical functioning (52.4 ± 5.3 vs. 54.2 ± 4.4, *p* < 0.001), role limitations owing to physical problems (51.3 ± 8.3 vs. 52.6 ± 7.7, *p* = 0.022), bodily pain (52.3 ± 8.1 vs. 53.9 ± 8.3, *p* = 0.003), mental health (38 ± 4.5 vs. 39.3 ± 4.3, *p* < 0.001), and PCS score (54.1 ± 6.9 vs. 55.7 ± 6.7, *p* < 0.001).

**Table 2:** Components of metabolic syndrome and SF-36 scores of participants

	Total		Men		Women	*P* value	
	*n*	%	*n*	*%*	*n*	%	
Components of metabolic syndrome							
Systolic blood pressure ≥ 130	287	31.7	166	47.8	121	21.6	< 0.001
Diastolic blood pressure ≥ 85	234	25.8	140	40.3	94	16.8	< 0.001
Triglyceride ≥150	202	22.3	119	34.3	83	14.9	< 0.001
HDL-C < 40 for male & < 50 for female	156	17.2	59	17.0	97	17.4	0.8922
Fasting glucose ≥100	172	19.0	86	24.8	86	15.4	0.001
Waist circumference ≥ 90 for male & ≥ 80 for female	417	46.0	142	40.9	275	49.2	0.015
History of diabetes mellitus							
No	741	81.8	280	80.7	461	82.5	0.769
Yes	28	3.1	12	3.5	16	2.9	
Missing	137	15.1	55	15.9	82	14.7	
History of hypertension							
No	714	78.8	256	73.8	458	81.9	0.004
Yes	78	8.6	42	12.1	36	6.4	
Missing	114	12.6	49	14.1	65	11.6	
History of dyslipidemia							
No	757	83.6	271	78.1	486	86.9	0.001
Yes	110	12.1	62	17.9	48	8.6	
Missing	39	4.3	14	4.0	25	4.5	
SF-36 (mean ± SD)							
Physical functioning	53.1	5.1	54.2	4.4	52.4	5.3	< 0.001
Role physical	51.8	8.4	52.6	7.7	51.3	8.3	0.022
Bodily pain	52.9	8.2	53.9	8.3	52.3	8.1	0.003
General health	47.3	9.2	47.8	9.6	47.0	9.0	0.195
Vitality	47.8	4.6	48.1	4.6	47.6	4.6	0.121
Social functioning	31.6	4.2	31.9	4.2	31.4	4.2	0.075
Role emotional	50.4	9.8	50.6	9.7	50.2	9.8	0.509
Mental health	38.5	4.5	39.3	4.3	38.0	4.5	< 0.001
Physical component summary (PCS)	54.7	6.9	55.7	6.7	54.1	6.9	0.001
Mental component summary (MCS)	38.0	5.0	38.1	5.0	37.8	5.0	0.417

Table 3 presents the results of regression analyses of the PCS scores. For all participants, men had significantly higher PCS scores than women (β = 1.353, *p* = 0.012); participants with more MetS components had significantly lower PCS scores (β = − 0.371, *p* = 0.043). Other significant factors associated with higher PCS scores including higher education level and higher physical activity level; current smokers had significantly lower PCS scores, and ex-smokers had higher PCS scores than non-smokers. For the different sexes, male participants with an education level of university/above had higher PCS scores than those who were illiterate or had an elementary school education (β = 17.02). Similarly, men with a high school education had higher PCS scores than those who were illiterate or only completed elementary school (β = 17.686). The table also shows that male participants with moderate and high physical activity had significantly higher PCS scores than those with low physical activity (β = 2.203, *p* = 0.005 and β = 3.370, *p* = 0.001, respectively). Interestingly, ex-smoker status was positively correlated with PCS scores whereas current smoker status was inversely correlated (β = 2.521 and − 1.721, respectively) in relation to non-smoker status. Among women, older age and number of components of MS were significantly associated with lower PCS score (β = − 0.114, *p* = 0.041 and β = − 0.542, *p* = 0.036, respectively); however, education and physical activity levels were not significantly associated with PCS scores.

**Table 3:** Multiple linear regression model for correlates of SF-36 PCS score

Covariates	Total		Males		Females	
	Beta	*P* value	Beta	*P* value	Beta	*P* value
Demographics						
Sex (male vs. female)	1.353	0.012	–	–	–	–
Age (year)	−0.081	0.051	−0.007	0.912	−0.114	0.041
Education level						
Illiterate/elementary school	Reference		Reference		Reference	
High school	3.43	0.047	17.02	< 0.001	−0.035	0.986
University and above	4.724	0.006	17.686	< 0.001	1.588	0.418
Marital status (married vs. others)	− 0.197	0.757	0.591	0.607	−0.382	0.62
No. of component of metabolic syndrome	−0.371	0.043	−0.116	0.645	−0.542	0.036
Physical activity level						
Low	Reference		Reference		Reference	
Moderate	0.897	0.076	2.203	0.005	0.101	0.878
High	2.09	0.001	3.37	0.001	1.22	0.137
Personal habits						
Smoking						
Current smoker vs. non-smoker	−1.675	0.02	−1.721	0.036	−1.084	0.421
Ex-smoker vs. non-smoker	2.451	0.014	2.521	0.019	2.393	0.276
Alcohol consumption (yes vs. no)	1.163	0.087	0.483	0.536	2.442	0.055

Table 4 presents the results of regression analyses of the MCS scores. For all participants, sex and number of MetS components were not significantly associated with MCS scores. However, participants with a high school-level education had significantly lower MCS scores than those who were illiterate or only completed elementary school (β = − 2.590, *p* = 0.042). Participants who were current smokers and consumed alcohol had significantly lower MCS scores than non-smokers and non-drinkers (β = − 1.394, *p* = 0.009 and β = − 1.173, *p* = 0.020, respectively). As for the different sexes, male participants with a high school education level had lower MCS scores than those who were illiterate or had an elementary school education (β = − 5.761. In addition, married men had significantly higher MCS scores (β = 2.837, *p* = 0.001), and males who reported alcohol consumption had significantly lower MCS scores (β = − 1.293, *p* = 0.03). Among female participants, current smoking was inversely correlated with MCS scores (β = − 2.322, *p* = 0.019).

**Table 4:** Multiple linear regression model for correlates of SF-36 MCS score

Covariates	Total		Males		Females	
	Beta	*P* value	Beta	*P* value	Beta	*P* value
Demographics						
Sex (male vs. female)	0.702	0.079	–	–	–	–
Age (year)	0.056	0.069	0.073	0.124	0.028	0.49
Education level						
Illiterate/elementary school	Reference		Reference		Reference	
High school	−2.59	0.042	−5.761	0.043	−1.727	0.231
University and above	−2.315	0.069	−4.778	0.091	−1.901	0.183
Marital status (married vs. others)	0.789	0.094	2.837	0.001	−0.058	0.918
No. of component of metabolic syndrome	0.121	0.369	−0.023	0.903	0.269	0.156
Physical activity level						
Low	Reference		Reference		Reference	
Moderate	−0.286	0.444	−0.611	0.302	−0.08	0.868
High	−0.769	0.098	−1.06	0.149	−0.335	0.578
Personal habits						
Smoking						
Current smoker vs. non-smoker	−1.394	0.009	−0.939	0.132	−2.322	0.019
Ex-smoker vs. non-smoker	0.614	0.402	0.800	0.327	0.776	0.63
Alcohol consumption (yes vs. no)	−1.173	0.02	−1.293	0.03	−0.784	0.401

## Discussion

In this study, after adjusting for covariates, we found that women but not men with more MetS components had significantly lower PCS scores. The results also showed significant sex differences in several dimensions, including education level, physical activity level, and smoking status for PCS scores; and education level, smoking status, and alcohol consumption for MCS scores.

Some studies have reported that declining physical HRQOL is accompanied by a greater number of MetS components in women but not in men [14, 21]. Katano et al. found that the number of MetS components was inversely associated with general health and positively associated with mental health in both sexes [15]. Although most previous studies have found that MS components are associated with worse HRQoL [15, 22], the reported associations between MetS components and HRQoL in the different sexes have been inconsistent.

A prospective study of middle-aged women in the United States showed that weight gain was associated with decreased physical functioning and vitality, as well as increased body pain, regardless of baseline weight [23]. In other recent studies, obesity was usually documented as being associated with poor HRQoL, not only in the physical but also in the mental domain [24, 25]. In our study, a trend indicating that higher numbers of MetS components were accompanied by lower physical health scores was found in women but not in men, indicating other mechanisms that are associated with this sex difference might deserve further investigation.

Although recent studies have demonstrated that dietary modification and enhanced physical activity may delay or prevent the transition from impaired glucose tolerance to type 2 diabetes mellitus and provide an adequate lifestyle for obese people [26], physical activity is probably not the most influential factor in MetS, or MetS may be affected by many factors. When adjustments for other lifestyle factors were made, we found significant associations between physical activity levels and physical health scores among men who participated in our study. Specifically, men who had insufficient physical activity had worse PCS scores than those with both moderate and high levels of physical activity; this finding was in line with previous studies [27, 28]. People with impaired HRQoL may also be less likely to participate in physical activity. On the other hand, it is possible that physical activity can improve HRQoL. However, because this was a cross-sectional research study, we could not determine any cause–effect relationship.

Some studies have found that physical activity most directly affects HRQoL through its impact on psychological well-being or emotional function [29]. Results from the Alameda County Study also suggest that people with low physical activity levels have a higher risk for depression compared with those who have higher levels of physical activity, independent of the presence of chronic conditions [30]. In our study, there was no difference in mental health scores between the sexes. More studies might be required to determine possible mechanisms explaining the relationship between physical activity and mental health.

In our study, we noted that male current smokers had significantly lower PCS scores whereas female current smokers had lower MCS scores than non-smokers. In addition, we noted that males who had quit smoking had better PCS scores than non-smokers. As we know, cigarette smoking plays a substantial role in the pathogenesis of numerous chronic diseases such as cardiovascular, cancer, lung, and other diseases [31, 32]. Thus, quitting smoking is a healthy behavior that can elevate not only HRQoL but also improve the environment, allowing others to feel more comfortable. Regarding alcohol consumption, the results in our study were line with those of several previous studies that also found that consuming alcohol was associated with poor HRQoL [33–37], especially for males [38]. The relationship between alcohol consumption and HRQoL is complex. Possible mechanisms included alcohol-related medical conditions, mental disorders, social problems, accidents, and violence. Our results suggest that maintaining healthy behaviors can lead to better HRQoL.

The significant positive association between high education levels and PCS score and the negative association between education levels and MCS score were found in males but not females. Sex differences in the associations between education level and PCS and MCS scores might be complicated. People with higher education levels might have better health knowledge, which could be helpful in maintaining healthy behaviors, disease prevention, and seeking professional medical treatment [39–41], all of which positively influence physical health-related quality of life. For mental health-related quality of life score, the negative associations might be related to stress from various aspects of daily life such as family issues, work competition, and so on. Males who were married had significantly higher MCS scores, which might be related to having better family supports.

The results of this analysis have some limitations. The analysis was cross-sectional in nature, such that making determinations about cause and effect is impossible. Furthermore, the sample in our study was local and too small to be representative of the entire population of Taiwan. In addition, mechanisms of the sex differences found in the associations between HRQoL and MS are unclear; these might be both biological and psychosocial. It is possible that sex variations in health-related behaviors (e.g., physical activity, smoking, alcohol consumption) may have many different kinds of impact on HRQoL outcomes. There are also differences in symptom presentation and management between men and women that could affect their perceptions of HRQoL. Thus, the sex differences in HRQoL require further study. However, to our knowledge, there have been few previous studies on the association between HRQoL and MS in Asian countries, especially in Taiwan. Our study was the first of its type to consider a middle-aged population in Taiwan, and the findings should allow us to better understand the health burden of MS among Taiwanese adults. A clearer finding of sex differences related to the association between MS and HRQoL will offer more information, to improve HRQoL of the population in the future.

## Conclusion

The results of this study showed that, after adjusting for covariates, women but not men with more MetS components had significantly lower PCS scores. The number of MetS components was not a significant factor of MCS scores between sexes. Furthermore, there were sex differences regarding age, education level, physical activity, and smoking status in association with PCS scores. For MCS scores, sex differences were found in education level, marital status, and habits of smoking and alcohol consumption. Further research should be conducted to investigate mechanisms of these sex differences.

## Abbreviations

BMI: Body mass index
HRQoL: Health-related quality of life
IPAQ-Taiwan: International physical activity questionnaire Taiwan version
IQOLA: International quality of life assessment
MCS: Mental component summary
MET: Metabolic equivalent of task
MetS: Metabolic syndrome
PCS: Physical component summary
SF-36 Taiwan: Short form-36 Taiwan version
